# Antioxidant Properties of Mushroom Mycelia Obtained by Batch Cultivation and Tocopherol Content Affected by Extraction Procedures

**DOI:** 10.1155/2014/974804

**Published:** 2014-07-10

**Authors:** Emanuel Vamanu

**Affiliations:** Department of Industrial Biotechnology, University of Agronomical Sciences and Veterinary Medicine Bucharest, Faculty of Biotechnology, 59 Marasti Boulevard, 011464 Bucharest, Romania

## Abstract

The determination of the antioxidant potential of lyophilized mushroom mycelia from 5 strains of the species *Pleurotus ostreatus* and *Coprinus comatus* (obtained by submerged cultivation in batch system) was analyzed as ethanolic extracts by evaluating ABTS and the hydroxyl scavenging activity, FRAP method, the chelating capacity, the inhibition of human erythrocyte hemolysis, and the inhibition of xanthine oxidase activity. The main compounds present in all extracts were determined by HPLC chromatography. Overall, results demonstrated that the biologically active substances content is modulated by the extraction method used. The most beneficial extract, characterized by determining the EC_50_ value, was that of *C. comatus* M8102, followed by *P. ostreatus* PQMZ91109. Significant amount of *α*-tocopherol (179.51 ± 1.51 mg/100 g extract) was determined as well as flavones such as rutin and apigenin. In the *P. ostreatus* PQMZ91109 extract, 4.8 ± 0.05 mg/100 g extract of tocopherol acetate known to play a significant role as an antioxidant in skin protection against oxidative stress generated by UV rays was determined. The various correlations (*r* ^2^ = 0.7665–0.9426 for tocopherol content) assessed and the composition of extracts in fluidized bed from the mycelia of the tested species depicted a significant pharmacological potential as well as the possibility of usage in the development of new functional products.

## 1. Introduction

Medicinal mushrooms are an effective alternative in the prevention and treatment of many modern diseases.* P. ostreatus* and* C. comatus *are used as a food source; however, due to their high content in therapeutic biomolecules,* P. ostreatus* and* C. comatus *are also a source of biologically active compounds. Fungal mycelia can easily be acquired in sufficient quantities by fermentation. Due to an increase in demand for the extraction of bioactive molecules in order to produce biologically active supplements, the determination of an effective and efficient method of extraction is thus required. The submerged cultivation method is an acceptable method; however increased efficiency is very important. The extractive efficiency depends mainly on the species used, on the nutrient sources in the culture medium, and on the cultivation parameters [1].

Mycelia extracts prevent free radical attack known to initiate membrane lipid peroxidation, resulting in an overall increased ability in defense against cellular malignization. Free radicals react with various molecules at the cellular level via oxidative stress and, as a result, bring about a perturbation of the normal cellular cycle. Free radicals are also known to accelerate the aging process, therefore disrupting various known natural defense mechanisms. Thus, taking supplements with such extracts is an alternative means against oxidative stress [2]. In addition, the presence of tocopherols is shown to be associated with the protection of low density lipoproteins (LDL) against oxidative stress. Oxidized LDL has been correlated with high plasma cholesterol levels [3]. Thus, the use of this mycelia extract may offer protection against the risk of developing cardiovascular disease, preventing atherosclerosis, and ultimately eliminating the risk of developing myocardial infarction.

Fungal fermentation in liquid medium ensures a high uniform quantitative biomass production as well as a high biological value, thus representing an alternative means to obtaining the various potential medicinal products. The fermentation technology has a role in maximizing the production of biomass and the level of biologically active components it contains [4].* In vitro* antioxidant activity has been correlated with phenolic components (i.e., amounts of anthocyanins and of tocopherols) [5]. The phenolic concentrations of the extracts depend on the species used, on the method used in obtaining the extracts (the process of extraction and conditioning), and on the evaluation method [6]. As a large amount of tocopherols was identified following the fluidized bed extraction from dried fruit bodies of the oyster mushroom (data not yet published), a similar amount is expected in the same type of extract from the mycelia of the* P. ostreatus* species. Thus, the aim of this study was to assess the antioxidant activity of lyophilized mycelia (submitted to extraction in fluidized bed) from 5 strains of* P. ostreatus* and* C. comatus* and to both correlate and identify the key molecules (by HPLC chromatographic analysis), which determine these antioxidative activities. The mycelium of each species was obtained by submerged cultivation in a batch system.

## 2. Materials and Methods

### 2.1. Chemicals

All chemicals and reagents were purchased from Sigma Aldrich GmbH (Steinheim, Germany). All other unlabelled chemicals and reagents were of analytical grade [7].

### 2.2. Microorganism, Media, Fermentations Conditions, and Fluidized Bed Extraction Process

Mushrooms belonging to* P. ostreatus* PQMZ91109,* Pleurotus ostreatus* PBS281009,* P. ostreatus* PSI101109,* P. ostreatus* M2191, and* C. comatus* M8102 were obtained from the collection of the Faculty of Biotechnology, Bucharest, Romania. The mycelia were kept in Nalgene cryotubes grown on barley grains, in 20% glycerol, at −80°C. The mycelia were revitalized on the medium of potato dextrose agar (PDA) at 4°C. The inoculum was prepared by growing the mushrooms on a LabTech rotary shaker at 150 rpm, for five days, at 25°C, in 500 mL Erlenmeyer flasks containing 250 mL of the culture medium containing 6.0 g glucose, 100.0 g malt extract, 20.0 g yeast extract, 1.0 g KH_2_PO_4_, and 0.5 g MgSO_4_  ×  7H_2_O, per liter. The medium was adjusted to a pH of 5.5 with 0.2 M NaOH [6, 7].

The second inoculum was performed in a 500 mL flask containing 300 mL of the medium after inoculating with 10% (v/v) of the first inoculum culture under the conditions described above. The fermentation medium (KH_2_PO_4_ 0.2%, CaSO_4_ 0.5%, MgSO_4_ 0.05%, Na_2_HPO_4_ 0.01%, and corn extract (dry substance 40%, as nitrogen source) 1% in 5% solution of corn starch) was inoculated with 10% (v/v) of the second inoculum culture and then cultivated in a 5-l New Brunswick BioFlo 310 bioreactor. Fermentations were conducted under the following conditions: temperature 25°C, aeration rate 1 vvm, agitation speed 150 rpm, pH 5.5–6, and working volume 4 L. The inoculum culture was then transferred to the fermentation medium and cultivated for 10 days [7].

The mycelium was recovered from the liquid medium by centrifugation at 4000 ×g (Centurion C2041 centrifuge) for 15 min. Next, the obtained mycelia were washed 3 times with distilled water and freeze-dried in an Alpha 1-2 LD freeze-dryer in the absence of a cryoprotective agent [6].

A quantity consisting of 50 g of the freeze-dried mycelia and 150 mL ethanol 70% was used to generate an extract using a fluidized bed extractor (fexIKA 200, IKA Labortechnik), after two extraction cycles. The alcohol extracts were concentrated in a rotary evaporator (Buchi R 210) with vacuum controller at 50°C, 175 mbar, and 200 rpm. The elected concentrated solution was freeze-dried in a Martin Christ Alpha 1-2 LD, to obtain the solid substance. The dried fractions were then redissolved in 80% ethanol to yield solutions containing 0.2–1.0 mg of extract per mL [8].

### 2.3. Antioxidant Activity Determinations

#### 2.3.1. ABTS (2,2′-Azino-bis(3-ethylbenzothiazoline-6-sulphonic Acid)) Radical Scavenging Activity

Radical scavenging assay ABTS radical cations are produced by reacting ABTS (7 mM) and potassium persulfate (2.45 mM) on incubating the mixture at room temperature in darkness for 16 h. The solution thus obtained was further diluted with phosphate buffered saline to give an absorbance of 1.000. Different concentrations of the extracts (0.2–1 mg/mL), 50 *μ*L, were added to 950 *μ*L of the ABTS working solution to give a final volume of 1 ml. The absorbance was recorded immediately at 734 nm with the Helios *λ* spectrophotometer. The percentage of inhibition was calculated with the following equation: % inhibition = [(Absorbance of control − Absorbance of test sample)/Absorbance control] × 100. Ascorbic acid was used as standard [9].

#### 2.3.2. Determination of Hydroxyl Radical Scavenging Activity

Quantities consisting of 0.2 mL of 0.1 mM FeSO_4_/0.1 mM EDTA*·*2Na, 0.2 mL 2-deoxyribose (10 mM), 0.2 mL sample (different concentration 0.2–1 mg/mL), and 1.2 mL phosphate buffer (0.1 M; pH 7.4) were mixed. After the addition of 0.2 mL H_2_O_2_ (10 mM), the mixture was incubated at 37°C for 4 h, and the reaction stopped by addition of a 1 mL trichloroacetic acid (2.8%) solution. Thiobarbituric acid/50 mM NaOH (1%; 1 mL) was then added and the mixtures heated at 100°C for 10 min, followed by rapid cooling and measurement of OD_532_ [10].

#### 2.3.3. Ferrous Ion Chelating Activity

To determine the Fe ion chelating ability, first, 1 mL of each polysaccharide (0.2–1 mg/mL) was mixed with 3.7 mL of ultrapure water, following which the mixture was reacted with ferrous chloride (2 mmol/L, 0.1 mL) and ferrozine (5 mmol/L, 0.2 mL) for 20 min. The absorbance at 562 nm was determined spectrophotometrically. EDTA was used as positive control. The chelating activity on the ferrous ion was calculated using the following equation: Chelating Activity (%) = [(Ab − As)/Ab] × 100, where Ab is the absorbance of the blank without the sample or ascorbic acid and As is the absorbance in the presence of the extract or EDTA [7, 11].

#### 2.3.4. Inhibition of Human Erythrocyte Hemolysis

The capacity to inhibit human erythrocyte hemolysis was based on the method described by Barros et al., 2007 [12]. Blood was obtained by harvesting from the first author. Blood tubes were immediately centrifuged at 3000 rpm for 10 min in a cooled Heidolff 320R centrifuge, with cooling at 9°C. The sediment was washed three times with 0.9% NaCl, and the reunited final sediments were brought into a 10% solution in 7.4 phosphate buffer [13]. The reaction mixture consisted of 0.1 mL of 10% human erythrocytes suspension, 0.2 mL of 200 mL 2,2′-azo-bis(2-amidinopropane) dihydrochloride, and 0.1 mL sample of extract (0.2–1 mg/mL). Test tubes were maintained at 37°C for 3 h. For dilution, 8 mL phosphate buffer pH 7.4 was added and each sample was centrifuged at 3000 rpm for 10 min. Finally, absorbance was read at 540 nm, and the inhibition of human erythrocyte hemolysis was calculated following the equation [(*A*_C_ − *A*_S_)/*A*_C_] × 100, where *A*_C_ represents the absorbance of the control sample without extract and *A*_S_ is the absorbance of the sample containing the extract. TBHQ was used as standard. The EC_50_ value (mg extract/mL), that is, the effective concentration at which the inhibition of human erythrocyte hemolysis is 50%, was obtained [14].

#### 2.3.5. Ferric Reducing Antioxidant Power (FRAP) Assay

The FRAP reagent was prepared by adding 2.5 ml of 10 mM TPTZ into 40 mM HCl. After dissolving TPTZ in HCl, 2.5 ml of 20 mM FeCl_3 _was added and 25 ml of 0.3 M acetate buffer pH 3.6. Then, approximately 3 ml of the FRAP reagent was added to 100 *μ*L of mushroom extract and 300 *μ*L of distilled water. The absorbance was measured at 593 nm against the blank after 4 minutes. FRAP value was calculated and expressed as mM Fe^2+^ equivalent (FE) per 100 g sample using the calibration curve of Fe^2+^ [15].

### 2.4. Xanthine Oxidase Inhibition Assay

Xanthine oxidase activity was determined by measuring the formation of uric acid from xanthine. All extracts were prepared in 50 mM phosphate buffer solution (pH 7.0). 2 mL of the sample was mixed with solution containing xanthine oxidase (2 mL, 0.4 U/mL) and xanthine (100 *μ*M). After incubating at room temperature (24°C) for 3 minutes, uric acid production was determined by measuring the absorbance at 295 nm. The inhibition percentage of xanthine oxidase activity was calculated according to the formula = [(*A*_control_ − *A*_sample_)/*A*_control_] × 100% [16].

### 2.5. Determination of Antioxidant Component

#### 2.5.1. Determination of Total Phenolic Content

The content of total phenols was determined by spectrophotometry, using gallic acid as standard, according to the method described by the International Organization for Standardization (ISO) 14502-1. Briefly, an aliquot of the diluted sample extract (1.0 mL) was transferred in duplicate to separate tubes containing a 1/10 dilution of Folin-Ciocalteu's reagent in water (5.0 mL). Then, a sodium carbonate solution (4.0 mL, 7.5% w/v) was added. The tubes were then allowed to stand at room temperature for 60 min before absorbance at 765 nm was measured against water. The content of total phenols was expressed as mg/g biomass. The concentration of polyphenols in samples was derived from a standard curve of gallic acid ranging from 10 to 50 *μ*g/mL (Pearson's correlation coefficient: *r* ^2^ = 0.9996) [6, 17].

#### 2.5.2. Determination of Total Flavonoids

Sample (0.25 mL of the extracts) was added to a tube containing distilled water (1 mL). Next, 5% NaNO_2_ (0.075 mL), 10% AlCl_3_ (0.075 mL), and 1 M NaOH (0.5 mL) were added sequentially at 0, 5, and 6 min. Finally, the volume of the reacting solution was adjusted to 2.5 mL with double-distilled water. The absorbance of the solution at a wavelength of 410 nm was detected using the Helios *λ* spectrophotometers. Quercetin is a ubiquitous flavonoid, present in many natural extracts, used as standard to quantify the total flavonoid content. Results were expressed in mg/g biomass [6, 18].

#### 2.5.3. Determination of *β*-Carotene and Lycopene

For *β*-carotene and lycopene determination, the dried ethanolic extract (100 mg) was vigorously shaken with an acetone-hexane mixture (4 : 6, 10 mL) for 1 min and filtered through Whatman number 1 filter paper. The absorbance of the filtrate was measured at 453, 505, and 663 nm. *β*-Carotene and lycopene content were calculated according to the following equations: lycopene (mg/100 mL) = − 0.0458 × *A*_663_ + 0.372 × *A*_505_ − 0.0806 × *A*_453_; *β*-carotene (mg/100 mL) = 0.216 × *A*_663_ − 0.304 × *A*_505_ + 0.452 × *A*_453_. The results are expressed as mg of carotenoid/g of extract [6, 12].

#### 2.5.4. Determination of Ascorbic Acid

Determination of ascorbic acid was determined by the method described by Barros et al., 2007. Content of ascorbic acid was calculated on the basis of the calibration curve of L-ascorbic acid, and the results were expressed as mg of ascorbic acid/g extract [12].

#### 2.5.5. Determination of the Total Quantity of Polyphenol Carboxylic Acids, Flavones, and Tocopherols 

Determination of the total quantity of polyphenol carboxylic acids, flavones, and tocopherols was determined by means of chromatography using high-pressure liquid chromatograph (HPLC) ELITE-LaChrom, with DAD detector, and presented in a previous study [5].

### 2.6. Statistical Analysis

All the assays for fermentation and antioxidant activity were assessed in triplicate, and the results were expressed as mean ± SD values of the three sets of observations (*P* < 0.05). The mean values and standard deviation were calculated using the EXCEL program from Microsoft Office 2010 package [7].

## 3. Results and Discussion

### 3.1. Mycelium Growth and Total Phenols and Flavonoids Accumulation in the Biomass

The maximum amount of biomass accumulation was achieved by maintaining optimal conditions of pH, temperature, and stirring. The profile of the growth curve showed a steady rollout after the eighth day with a maximum following the tenth day of submerged fermentation in a batch system. It was also observed that, in the biomass samples taken during the fermentative processes, the accumulation of the antioxidant compounds was directly proportional to the increase in biomass amount (data not shown) with the exception of* C. comatus *M8102 (45.37 ± 3.43 g/L/day biomass) and* P. ostreatus* PSI101109 (16.63 ± 0.77 g/L/day biomass), which showed a decrease in the total amount of phenols and flavonoids with entry into the stationary phase (Table 1). The decrease did not exceed 10% and remained constant until the end of the fermentative process. This observation was also supported by the carbon source depletion. The maximum growth rate was calculated for* P. ostreatus *PQMZ91109 (0.56 ± 0.09 h^−1^). For the other species, the determined differences are the result of an exponential growth phase of the mycelia which stretched over a longer period of time.

The gradual depletion in carbon source and the change in ratio between amount of carbon and nitrogen sources are a possible explanation as this behavior is typical of the cultivated species. This behavior occurred when the carbon source was depleted in favor of the nitrogen source resulting in a direct reduction in the growth rate of the mycelia and of the sizes; however, the density of mushroom pellets is increased. Thus, there was an increase in total amount of phenols and flavonoids present in the biomass produced by batch fermentation compared with the cultivation in stirred flasks (inoculum), in which growth was at least 20% in* C. comatus* M8102. The accumulation of these secondary metabolites can also be explained as a mushroom response to the stress as determined by the inversely proportional decrease of the amount of carbon source in the case of batch cultivation. Such a behavior has been previously demonstrated both in plants and in other fungi (*Russula griseocarnosa*) [1].

The accumulation of secondary compounds showed much higher values than the cultivation in other conditions (in Erlenmeyer flasks), ranging between 20% and 50%. The highest amount was found in the mycelium of* P. ostreatus* PSI101109 (98.60 ± 1.85 mg/g biomass). The least productive species was* P. ostreatus* PBS281009 (35.40 ± 6.75 mg/g biomass), with no more than 65% of the other tested species (Table 1). The flavonoids are the most important group of phenolic compounds, representing over 50% of the total registered amount with the exception noted by* C. comatus* M8102 with a rate of only 34%. If we consider the accumulation of biomass in the course of the batch fermentative process, the correlation with the total amount of polyphenols showed positive correlations (*r* ^2^ = 0.514–0.689) which also corresponded to the amount of determined flavonoids (*r* ^2^ = 0.679–0.861), according to species. Thus, it has been shown that the synthesis of these secondary metabolites occurs during different periods of the mycelia growth phases in a batch system. A marked accumulation of flavonoids was also noted toward the end of the exponential phase of growth and with entry into the stationary phase. This behavior has been observed in both* P. ostreatus* PSI101109 and* P. ostreatus* PBS281009, where the amount of flavonoids in the total phenolic compounds was highest, by at least 70%.

The quantities of phenolic compounds in the obtained extracts were lower than expected possibly due to their concentration levels being below the limit of detection of the high performance liquid (HPLC) chromatographic method used. An additional problem may be due to extraction procedure used, which favors the extraction of other active compounds (tocopherols), possibly as a result of a significant decrease in the amount of total phenolic compounds. This is the first published investigation known to show the effect of ethanol extraction in fluidized bed on the phenolic composition of lyophilized mycelium extract. Even though the presence of these molecules is indicated by the Folin Ciocalteu method, the concentrations may be influenced by interaction with other active ingredients, as has been shown by recent studies [19]. This observation has also been depicted in the case of fluidized bed extraction from the fruit body of the same species, as well as some wild ones such as* Tuber melanosporum*,* Marasmius oreades,* or* Craterellus cornucopioides*—data yet to be published.

The main phenolic acids determined were homogentisic acid (0.14 ± 0.001–35.18 ± 0.4 mg/100 g of extract) and gallic acid (0.82 ± 0.01–16.21 ± 0.16 mg/100 g of extract) compounds that were found present in all extracts from the mycelium. In addition, a small amount of chlorogenic acid in the* P. ostreatus* M2191 mycelium extract was also detected (Table 2). A significant number of flavones were also identified, indicative of the presence of rutin in* C. comatus* M8102 extract and also the lack of catechin, except for the* P. ostreatus* PQMZ91109 mycelium extract. Another novel finding was the presence of apigenin (0.034 ± 0.00 mg/100 g extract) in the* C. comatus* M8102 extract, which is responsible for the inhibition of membrane lipid peroxidation [20]. An additional finding as a result of the chromatographic analysis was the identification of pyrogallol in the* P. ostreatus* PBS281009 mycelium extract, which has not been identified for this species in previous studies [19].

While most studies focus mainly on the phenolic profile, the tocopherol composition has not been analyzed as much in medicinal mushroom mycelia extracts. As such, the fluidized bed extraction process of the present study resulted in the identification of significant amounts of *α*-tocopherol, with the greatest significant amount determined in the* C. comatus* M8102 extract. A third novel finding of this study was the presence of tocopherol acetate in the extract of* P. ostreatus *PQMZ91109. Tocopherol acetate is the ester moiety formed between acetic acid and *α*-tocopherol (4.8 ± 0.05 mg/100 g of extract), which is known for its inhibitory effect on tyrosinase, which has an important physiological significant role in controlling melanin production.

In addition to the phenolic compounds, following the extraction in fluidized bed from the mycelium, a significantly similar amount of ascorbic acid was obtained in the analyzed extracts (Table 2). A similar trend was observed for the accumulation of *β*-carotene and lycopene for the four species of* Pleurotus*. In the* C. comatus* M8102 mycelium extracts, an amount of *β*-carotene determined to be 88.5% higher was identified, and 3 times more lycopene was also determined in comparison to the* P. ostreatus* mycelia extracts. Results depict the specific characteristic behaviors of the two species in the case of cultivation in the bioreactor, under steady conditions. In this manner, the fermentative batch process may be used in order to obtain a mycelium rich in the most important secondary metabolites of biological activity.

### 3.2. Evaluation of Antioxidant Activity of Extracts in Fluidized Bed from Mycelium Obtained by Batch Cultivation

The evaluation results of the antioxidant activity of lyophilized mycelia of all five fungal strains are presented as EC_50_ values in Table 3. The evaluation of free radical scavenging activity was performed by using ABTS and hydroxyl radicals. Compared to other methods used to assess free radical activity, the ABTS scavenging activity assessment results in greater accuracy. As both the hydrophilic antioxidant component and the lipophilic components of the extract are known to react in the assessment [21], the free radical scavenging activities were as follows (in increasing order):* C. comatus* M8102 <* P. ostreatus* PSI101109 <* P. ostreatus* M2191 <* P. ostreatus* PQMZ91109 <* P. ostreatus* PBS281009. Hydroxyl radicals are among the most reactive of reactive oxygen species and can cause increased oxidative damage to DNA, lipids, and proteins [6]. Thus, for the hydroxyl radical scavenging activity, the increasing order of EC_50_ value was as follows:* P. ostreatus* PBS281009 <* P. ostreatus* M2191 <* C. comatus* M8102 <* P. ostreatus* PQMZ91109 <* P. ostreatus* PSI101109.

Chelating agents are known to prevent the generation of oxyradicals. For that purpose, a chelating method was used to evaluate antioxidant activity [22]. Human erythrocytes represent an ideal* in vitro* model which is very useful due to their membranes which contain polyunsaturated fatty acids which are extremely susceptible to free radical attack following the decomposition of AAPH [23]. The direct relationship therefore between both methods is a precise indicator of the ability to prevent lipid peroxidation, expressed as peroxyl radicals scavenging activity. In both cases, the increasing order was as follows:* C. comatus* M8102 <* P. ostreatus *PQMZ91109 <* P. ostreatus *PSI101109 <* P. ostreatus* M2191 <* P. ostreatus* PBS281009.

The FRAP method is an accurately known method used to quantify the antioxidant potential of an extract. The methodology is based on the reaction of the extracts of antioxidant components with inactivated oxidants. The reducing power exerted by the antioxidant molecules from the analyzed extract is associated with the interruption of free radical formation [24]. The results obtained as EC_50_ values demonstrated a ferric ion reducing capacity of 25% higher on average, compared with the standard (ascorbic acid). These values were up to 10 times lower than the methanol extracts of edible wild species (*Russula nigricans*,* Amanita rubescens var. Rubescens*,* P. dryinus,* or* Leccinum scabrum*) [25].

For the assessments of such extracts in the prevention and/or support of conventional treatments to inhibit the inflammatory processes caused by the formation of uric acid, the inhibition of xanthine oxidase is a widely accepted* in vitro *model. For evaluation of the mycelium, the inhibition of formation of uric acid (a substance which causes the disease gout) grew and produced the following results:* C. comatus* M8102 >* P. ostreatus* PQMZ91109 >* P. ostreatus* M2191 >* P. ostreatus* PSI101109 >* P. ostreatus* PBS281009. Thus, due to the biological activity of the mycelium extract, it is recommended as an additive or as part of the composition of some products which protect the liver, thus fulfilling also a possible additional detoxifying role by increasing the efficiency of the removal function, favoring the inhibition of excess uric acid production. The presence of apigenin in the extract of* C. comatus* M8102 can confirm probable anti-inflammatory effects of mycelium. This flavonoid is known to inhibit the physiological process of formation of uric acid [26].

The bioreactor cultivation of mycelia is a reproducible method in obtaining a valuable substrate from extracts with high biological activity. The mycelia contain antioxidant compounds that differ from those present in the fructification body of the fungus. An example is the presence of intracellular and extracellular polysaccharides significantly contributing to an increase in the biological activity of the obtained mycelia. As the molecular weight is directly proportional to the bioactivity, the fermentation process is the most important in the synthesis of polysaccharides resulting in molecular weight as high as possible. This occurs with a depletion of the carbon source and the entry into the stationary phase. Thus, as a result, it was determined that while the proliferation of the mycelia grown in batch culture is related to the carbon source, the amount of antioxidant compounds from the mycelia is to a larger extent related to the source of nitrogen and to the maintenance of an appropriate pH level [7]. A smaller size of the mushroom pellets resulted in a greater accumulation of compounds with antioxidant activity. This observation was supported by the results shown in Table 1, for the strains* C. comatus* M8102,* P. ostreatus* M2191, and* P. ostreatus* PQMZ91109. An increase in the amount of the secondary compounds, of approximately 35%, was registered compared to the ethanol extracts in fluidized bed from the fruiting body of the* P. ostreatus* species [27]. If the rheological properties of the medium can represent a direct indication of the synthesis of a specific metabolite such as exopolysaccharides, the morphological aspect is significant enough to achieve mycelia rich in functional compounds. Changing the parameters of the fermentative process by increasing the stirrer speed does not result in increased efficiency of production of such compounds in the mycelia. Moreover, only a decrease in the amount of biomass was noted, with a direct effect on its composition [7].

Freeze drying is the most effective in retaining the maximum amount of compounds with antioxidant activity in an extract. As such, freeze drying was therefore used to obtain a valuable substrate for the later stage of the extraction. The ethanol and water mixture is believed to be the solvent mix that ensures appropriate extraction efficiency, expressed as the amount of antioxidants/g of substrate. This was shown in the case of* Agaricus brasiliensis* mycelium [28]. By using the extraction in fluidized-bed from the lyophilized mycelia of* P. ostreatus* and* C. comatus,* an increase in antioxidant potential of the lyophilized extract was determined. A comparison was made with the mycelia obtained by submerged cultivation in Erlenmeyer flasks and submitted to a simple extraction (also a lyophilized mycelium) [6]. Extraction in fluidized bed favored the presence of tocopherols, which represents a novelty, compared to most studies in which various phenolic compounds predominate. Results may lead to the possibility of modulating the composition of the active compounds in the final extract by the extraction method used. This possibility is novel as simple extractions in previous studies have not led to similar results for the same species [29]. The significance in our findings is supported both by the determined amount and by the localization within the cell membrane of mainly *α*-tocopherol.

Extracts from the mycelia of* P. ostreatus* and* C. comatus *showed relatively similar capabilities of free radical scavenging and of ferric ion chelation; however a lower capacity (EC_50_ value greater on average by 60%) for the protection of lipid peroxidation by the peroxyl radicals was noted. Compared with the extracts from* Leucopaxillus giganteus*,* Sarcodon imbricatus,* and* Agaricus arvensis,* the mycelia of the two mushrooms submitted to extraction in fluidized bed, a product with superior capabilities for the protection of the erythrocyte membrane against radical attack generated by AAPH, which may cause hemolysis, were determined [12].

These antioxidant capabilities are all considered to mainly be linked with the phenolic components of the extracts, resulting in significant medicinal efficacy, thus supporting the idea of using such compounds as active ingredients in functional products. The data is in contradiction with the effects of the herb* Hypericum perforatum* extracts which contain phenolic compounds and flavonoids with low affinity for the peroxyl radicals, thus validating the significance of the composition of the active components (mainly phenolic compounds) that are involved in the scavenging effect [30]. Besides the significant antioxidant efficacy, the mycelium extracts in fluidized bed demonstrated an anti-inflammatory effect. This property is also directly expressed by the total amount of phenolic acids and flavonoids, as noted bythe* in vitro* inhibition of xanthine oxidase by* C. comatus* M8102 extracts.

The types and amounts of phenolic compounds varied with the same culture conditions of the species; however, similar antioxidant activity was observed. It can therefore be concluded that other components may be involved in these antioxidative properties as mentioned in previous studies [7, 31]. According to published data with respect to the relationships between the concentration of phenolic compounds and the antioxidant activity there is much controversy. Moreover, antioxidant properties have also been shown to be influenced by the content of tocopherols, anthocyanins, and carotenoids compounds [32].

A positive correlation expressed by* in vitro* analysis between the amount of biologically active compounds and the different antioxidative properties of each separate extract was determined. With respect to the *α*-tocopherol content, the *r* ^2^ value (0.7665–0.9426) was greatest for the inhibition of xanthine oxidase, the inhibition of erythrocyte hemolysis, and the FRAP method for the evaluation of the reducing power (*P* < 0.0001). As for the inhibition of the two species of free radicals, the value of the correlation index with the tocopherol content was moderate.

The polyphenolic content had a medium degree of correlation, with the largest correlation related to the capacity to inhibit the erythrocyte hemolysis (*r* ^2^ = 0.476–0.9773). The greatest correlations *r* ^2^ were expressed for the species* C. comatus *M8102,* P. ostreatus* PQMZ91109, and* P. ostreatus* PSI101109 (*P* < 0.0001). With regard to the inhibition of xanthine oxidase, the FRAP method as well as the ability of chelation and the correlations with respect to the polyphenolic content were moderate. The ability to inhibit free radical species in pyrogallol of* P. ostreatus* PBS281009 was determined to be highly correlated. Overall, pyrogallol presence to the expression does not exceed 50% of the antioxidant potential. A similar behavior was also observed in* C. comatus* M8102 when the rutin and apigenin content were also considered. An increase of the *r* ^2^ value was also observed for the inhibition of hemolysis and xanthine oxidase and for the chelating capacity. This activity was also determined for the strains* P. ostreatus* PQMZ91109 and* P. ostreatus* PSI101109, where myricetin and catechin were present (*P* < 0.004).

In conclusion, choosing the appropriate extraction procedure allowing for the modulation of the biological value of the extract allows for new and improved technological implications. This is the first study known to evaluate the antioxidant capacity of a fluidized bed extract from mushroom mycelium. Regardless, the current results do not invalidate the pharmacological value of such preparations in alleviating the oxidative properties. The relevance of these* in vitro* results must be supported by future* in vivo* studies. Moreover,* in vivo* studies should be performed in the determination of antioxidant capacity and level of assimilation after passage through the human gastrointestinal tract.

## Figures and Tables

**Table 1:** Biomass production and total phenols and flavonoids accumulation in the submerged culture of mushrooms mycelia after 10 days of batch fermentation.

Mushroom species	Biomass productivity (g/L/day)	Total phenols (mg/g biomass)	Total flavonoids (mg/g biomass)	Product yield (g biomass/g carbon source)	Maximum growth speed (h^−1^)
*P. ostreatus* PQMZ91109	22.50 ± 1.50	63.40 ± 4.30	34.35 ± 1.67	45.05 ± 1.69	0.56 ± 0.09
*P. ostreatus* PSI101109	16.63 ± 0.77	98.60 ± 1.85	69.75 ± 3.52	38.04 ± 1.51	0.74 ± 0.01
*P. ostreatus* PBS281009	35.16 ± 1.71	35.40 ± 6.75	28.00 ± 5.70	50.22 ± 2.40	0.63 ± 0.12
*P. ostreatus* M2191	26.25 ± 0.81	70.00 ± 3.91	45.00 ± 2.89	29.16 ± 0.88	0.65 ± 0.05
*C. comatus *M8102	45.37 ± 3.43	72.60 ± 8.67	24.75 ± 1.28	37.80 ± 2.93	0.59 ± 0.1

**Table 2:** The main compounds with antioxidant effect identified in the fluidized bed extracts of lyophilized mycelium.

Compound (mg/100 g extract)	*P. ostreatus* PQMZ91109	*P. ostreatus* PSI101109	*P. ostreatus* PBS281009	*P. ostreatus* M2191	*C. comatus* M8102
Gallic acid	9.39 ± 0.1	16.21 ± 0.16	0.82 ± 0.01	10.56 ± 0.11	11 ± 0.1
Homogentisic acid	17.78 ± 0.2	35.18 ± 0.4	0.14 ± 0.001	10.93 ± 0.1	7.96 ± 0.08
Chlorogenic acid	—	—	—	0.0436 ± 0.0004	—
Catechin	0.67 ± 0.007	—	—	—	—
Rutin	—	—	—	—	0.19 ± 0.002
Myricetin	1.6 ± 0.02	1.32 ± 0.01	—	—	—
Apigenin	—	—	—	—	0.034 ± 0.00
Pyrogallol	—	—	64.67 ± 0.6	—	—
Ascorbic acid	0.94 ± 0.02	0.93 ± 0.04	0.93 ± 0.01	0.92 ± 0.02	0.94 ± 0.32
*β*-Carotene	0.02 ± 0.01	0.03 ± 0.02	0.02 ± 0.002	0.03 ± 0.002	0.13 ± 0.09
Lycopene	0.01 ± 0.004	—	0.01 ± 0.002	0.03 ± 0.001	0.09 ± 0.05
*α*-Tocopherol	14.52 ± 0.15	23.4 ± 0.2	30.47 ± 0.35	24.56 ± 0.56	179.51 ± 1.51
Tocopherol acetate	4.8 ± 0.05	—	—	—	—

**Table 3:** The antioxidant activity (expressed as EC_50_ value in mg/mL) of the extracts of lyophilized mycelium.

Mushroom species	ABTS scavenging activity	Hydroxyl scavenging activity	Chelating effect	AAPH	FRAP	XO
*P. ostreatus* PQMZ91109	0.63 ± 0.02	1.65 ± 0.04	0.17 ± 0.05	2.63 ± 0.13	1.76 ± 0.05	0.9 ± 0.21
*P. ostreatus* PSI101109	0.25 ± 0.01	1.48 ± 0.07	0.22 ± 0.02	2.65 ± 0.20	1.51 ± 0.04	1.8 ± 0.65
*P. ostreatus* PBS281009	1.78 ± 0.04	4.54 ± 0.01	3.67 ± 0.01	6.96 ± 0.28	4.77 ± 0.17	2.45 ± 0.54
*P. ostreatus* M2191	0.45 ± 0.11	2.20 ± 0.20	0.64 ± 0.01	4.15 ± 0.24	3.77 ± 0.04	1.41 ± 0.22
*C. comatus *M8102	0.10 ± 0.05	1.87 ± 0.03	0.12 ± 0.03	2.27 ± 0.15	0.72 ± 0.01	0.78 ± 0.08
